# Challenging or less challenging oral diadochokinetic tasks—what works best in Huntington disease? A cross-sectional study

**DOI:** 10.1007/s00415-025-13310-x

**Published:** 2025-10-15

**Authors:** Wiebke Hannemann, Lukas Stahuber, Tomas Kouba, Katrin S. Lindenberg, Daniel Rapp, Jan Lewerenz, Hans-Jürgen Huppertz, Tereza Tykalova, Jan Rusz, G. Bernhard Landwehrmeyer, Alzbeta Mühlbäck

**Affiliations:** 1 Department of Neurology, Huntington Center Ulm (HCU), Ulm University, Oberer Eselsberg 45, 89081 Ulm, Germany; 2 Department of Circuit Theory, Faculty of Electrical Engineering, Czech Technical University in Prague, Prague, Czech Republic; 3 Swiss Epilepsy Clinic, Klinik Lengg, Zürich, Switzerland; 4 Huntington Center South, Kbo-Isar-Amper-Klinikum, Taufkirchen (Vils), Germany; 5 Department of Neurology and Centre of Clinical Neuroscience, First Faculty of Medicine, Charles University and General University Hospital, Prague, Czech Republic

**Keywords:** Acoustic analyses, Hyperkinetic dysarthria, Huntington disease, Oral diadochokinesis, Quantitative digital biomarker

## Abstract

**Background:**

In Huntington disease (HD), speech alterations are common and may emerge before onset of chorea. Slow and irregular motion rates, i.e. altered oral diadochokinesis (oDDK), are a distinctive feature. This study investigated oDDK using alternating (AMR) and sequential motion rate (SMR) tasks in manifest HD and explored the impact of antidopaminergic medications (ADM).

**Methods:**

Speech samples were acquired from 30 healthy controls (14 men; 27–78 years¸ age- and gender-matched to HD subjects) and 35 individuals with early-to-moderate HD (18 men; 22–76 years) phenotyped using standardized scales (UHDRS’99) and MR imaging to estimate disease severity. Acoustic analysis was used to quantify rate and regularity of oDDK. In an exploratory subgroup analysis, the impact of ADMs on oDKK was explored by comparing patients with and without ADMs (HD-ADM: n = 16; 8 men; 22–76 years; HD-nADM; n = 19; 10 men; 28–61 years).

**Results:**

HD patients were slower and more irregular in AMR and SMR tasks (p < 0.001) compared to controls. Analyses using area under the receiver-operating characteristic curve (AUC) showed the best characteristics for AMR (AUC = 95.0%). oDDK parameters correlated with measures of motor, cognitive and functional impairment and striatal atrophy. Patients on ADMs showed slower motion rates in both tasks (AMR p = 0.021; SMR p = 0.026), but unchanged regularity.

**Conclusions:**

Decreased AMR performance alone captured early motor impairment accurately, reflects disease severity and is ADM-sensitive. Therefore, objective acoustic analysis of AMR performance is a simple measure which may serve as read-out to monitor disease progression, e.g. in clinical trials.

**Supplementary Information:**

The online version contains supplementary material available at 10.1007/s00415-025-13310-x.

## Introduction

Huntington disease (HD) is a hereditary neurodegenerative disease characterized by a triad of motor, psychiatric and cognitive signs [1] caused by an unstable Cytosine-Adenine-Guanine (CAG) repeat expansion mutation in the huntingtin (*HTT*) gene [2]. The brunt of the neuropathological alterations are observed in basal ganglia [3] and the extent of striatal atrophy is used to grade the severity of HD neuropathologically [4].

Speaking is a complex motor activity that requires the interplay of multiple subcortical and cortical areas [5, 6]. Speech is impaired early in the disease process of HD with quantifiable changes in speech parameters appearing before the emergence of diagnostic motor signs [7–10]. In HD, speech dysfunction is a hyperkinetic dysarthria with specific features such as imprecise consonants, prolonged intervals, variable rate, monopitch, harsh voice, inappropriate pauses, distorted vowels and excessive loudness variations [11].

Diadochokinesis (DDK) of the upper limbs is a key component of the neurological examination included in the Unified Huntington’s Disease Rating Scale (UHDRS) [12]. DDK may also be measured quantitatively using different devices [13]. A previous study using such a device in drug-naïve HD patients showed altered performance of rapid alternating movements, including simple and complex motion rates [13]. In addition, oral DDK (oDDK) is a key element in motor speech assessments, measuring the capabilities and limitations of speech articulators [11, 14]. Oral diadochokinetic performance is quantified by two primary metrics: the oDDK rate, which evaluates articulatory speed through the number of syllables produced per second, and the oDDK regularity, which assesses the consistency of syllable timing using the standard deviation between successive syllables [15]. In HD, both slow and irregular oDDK patterns have been observed [16]. Two widely used oDDK tasks are an alternating motion rate (AMR) task, i.e., a rapid repetition of single syllables (/ta/), and a sequential motion rate (SMR) task, i.e., a rapid repetition of a syllable sequence (/pa/-/ta/-/ka/). SMR is a more challenging task that requires a higher degree of motor planning [11] due to the need to rapidly alternate between different articulatory positions. This higher task complexity of SMR is evident in healthy speakers [17]. As HD progresses, the range of cognitive dysfunctions broadens and typically includes increasing deficits in sequential planning [18], and thus should result in even more pronounced deficits in SMR. Prior studies showed slower oDDK rates and higher variation of syllabic pace in premanifest [10, 16, 19] and manifest HD [14, 20, 21] compared to controls. Furthermore, a small-scale longitudinal study in premanifest HD mutation carriers demonstrated that repetition of single and pairs of syllables significantly changed over time [22]. However, there is only preliminary evidence demonstrating a correlation between oDDK parameters and the extent of motor and cognitive impairment [21] and only few data correlating declining performance in oDDK tasks to biological severity of HD [22]. In addition, prior studies are limited by small sample sizes [14, 20, 21], and undefined [20] or rater-based measures to estimate disease severity [14]. Finally, SMR performance in early-to-moderate stages of HD is largely unexplored [10] and a direct comparison of AMR and SMR tasks in HD is missing, with the exception of a pilot study suggesting a better effect size for SMR compared to AMR [16]. However, a comparative study exploring the diagnostic sensitivity of AMR vs. SMR in manifest HD patients is missing.

Currently, no disease modifying treatment is established for HD [23]; current clinical management is limited to symptomatic pharmacological and non-pharmacological treatment [24]. Antidopaminergic medication (ADM) is often used to reduce chorea and treat behavioural abnormalities [25]. ADMs may cause side effects including slowed voluntary movements [26, 27], tardive dyskinesia [28], and may impair cognition [25]. In terms of speech, ADMs may alter articulatory functions such as loudness, pitch and speech timing [29] and may reduce phonation time, syllable repetition rate and syllable repetition regularity [21]. To date, data on the impact of ADM on oDDK in HD are very limited.

Primarily, this cross-sectional study aims to systematically assess and compare the accuracy of AMR and SMR tasks in detecting alterations in oDDK in manifest HD patients. An additional primary aim was to compare oDDK between patients with manifest HD and controls. A secondary, exploratory aim of the study was to investigate the relationship between quantitative measures of oDDK and indicators of disease severity, including cognitive performance, functional capacity, CAP score, and MRI volumetry. Lastly, another exploratory secondary aim was to explore the effects of ADMs on oDDK in manifest HD, including the investigation of potential dose dependencies.

## Methods

### Study population

We recruited HD patients with clinically diagnosed and genetically confirmed HD (≥ 36 CAG repeats in one of the *HTT* alleles) and healthy controls (HC) at the Huntington Centre Ulm (HCU), Department of Neurology, Ulm University (between January 2020 and November 2021). The study was approved by the Ethics Committee of the Ulm University, Germany (approval number: 381/18) and was conducted in accordance with the Declaration of Helsinki and its later amendments. All participants gave written informed consent prior to enrolment. Exclusion criteria for participants in both groups were (1) not being a German language speaker and (2) having a disorder, unrelated to HD, interfering with speech and/or articulation (e.g., neurological, respiratory and pulmonary disorders) or (3) marked cognitive impairment interfering with study conduct.

For all participants demographics, medical history, and current pharmacotherapy were recorded along with the results of a physical as well as a neurological and neuropsychological examination; the HD phenotype was assessed using the UHDRS’99 [12] and included the total motor score (TMS; range = 0–124; higher scores indicate more severe motor impairment), a cognitive score derived from the UHDRS cognitive battery, including the Symbol Digit Modalities Test (SDMT), and Total Functional Capacity (TFC; range between 1 and 13; lower scores indicating more severe functional limitations). For disease staging, the HD-ISS staging system [30] (stage 0: asymptomatic; stage 1: biomarker only; stage 2: clinical signs and symptoms; stage 3: functional impairment) was employed. In addition, the Shoulson and Fahn staging system [31] based on TFC scores was used [stage I (13–11), stage II (10–7), stage III (6–3), stage IV (2–1) and stage V (0)]. To assess biological burden, we used a product of excess CAG repeats and age, CAP100, as defined by Warner et al. [32].

### Speech examination

Speech was recorded with a head-mounted condenser microphone (Shure Beta 53, Shure Inc, Niles, IL, USA) placed approximately 5 cm from the subject’s mouth in a quiet room with a low ambient noise level [33]. The speech recordings were sampled at 48 kHz with 16-bit resolution. The speech recordings were conducted by trained investigators following the assessment protocol [33]. For the AMR task, the participants were asked to repeat the syllable /ta/ as quickly as possible for at least 5 s in one breath. The syllable /ta/ was preferred over /pa/ because it best reflects tongue movement and previous studies have shown that tongue function in dysarthria is significantly more impaired than lip function [34]. For the more challenging SMR task, participants were instructed to continuously repeat the syllables /pa/-/ta/-/ka/ as accurately and quickly as possible in one breath. It was aimed to achieve at least seven, but ideally twelve repetitions of the syllable triple in one breath. Both, AMR and SMR tasks were performed twice.

### Acoustic speech analysis

For both AMR and SMR tasks, oDDK performance was quantified by calculating two primary metrics: the oDDK rate and oDDK irregularity. oDDK rate reflects the speed of articulation and was calculated as the number of syllable vocalizations per second. oDDK irregularity assesses temporal irregularity of syllable repetition and was computed as the standard deviation of time gaps between successive syllables. Details on algorithms behind the calculation of oDDK measures have been reported previously [35]. Data for each participant obtained via two vocal task runs were averaged to ensure greater stability of speech assessment [33]. All analyses were performed in MATLAB® (MathWorks, Natick, MA).

### Impact of antidopaminergic medication (ADM)

To evaluate the impact of ADMs on oDDK performance, HD patients were divided into a subgroup on ADMs (HD-ADM) and a subgroup not receiving ADMs (HD-nADM). To investigate dose-dependency, the HD-ADM group was stratified into a low (HD-ADM_low_) and high dosage group (HD-ADM_high_) by an experienced neurologist (for detailed information see Supplement). Moreover, chlorpromazine equivalents (CPZ) were calculated for each ADM thus normalizing dosages of different ADMs [36], although there is no generally accepted gold standard for calculating equivalent doses of ADMs [37].

### Magnetic resonance imaging

As an optional component, the study protocol included the acquisition of magnetic resonance imaging (MRI) data as biomarker of disease severity. In principle, both participant groups were given the opportunity to undergo MRI within the study. In this study cohort, 19 out of 35 manifest HD patients consented to the additional MRI data collection. MRI data was collected within a time frame of 99 days on average (*SD* = 150) from voice recording and was obtained using a state-of-the-art 3 Tesla (3T) whole-body MRI scanner (Magnetom Prisma, Siemens Healthcare GmbH, University Hospital Ulm, Germany). Parameters of primary interest were volumetric measures of predefined brain regions known to be altered in HD, namely the striatum, including the caudate and putamen. Volumetric measures were obtained from three-dimensional T1 weighted (3D-T1) structural MRI images using an atlas-based volumetry (ABV) method [38, 39], which is an objective and fully automated volumetric analysis method using the Statistical Parametric Mapping 12 software (SPM12, Wellcome Trust Centre for Neuroimaging, London, UK; www.fil.ion.ucl.ac.uk/spm) on MATLAB® (R2019b, MathWorks Inc., Natick, MA, USA) and predefined masks for e.g., subcortical structures derived from the Harvard–Oxford Atlas (HOA [40]). For a detailed description of the ABV method please refer to Huppertz et al. [38, 39].

### Statistical analysis

Repeated measures analysis of variance (RM-ANOVA) with GROUP (HD vs. HC) as between-group factor and TASK (AMR vs. SMR) as within-group factor as well as their interaction effect GROUP X TASK was used to assess differences between groups (HD vs. HC) and between oDDK tasks (AMR vs. SMR). For post-hoc analyses, pairwise t-tests were applied to compare the performance in both oDDK tasks (AMR vs. SMR) and for each group (HD vs. HC). Prior to these analyses, data were tested for normal distribution by visual inspection of QQ-Plots and histograms as well as by using the Shapiro-Wilks test and for homogeneity of variance using the Levene-Test. In case of violation of the normality assumption, non-parametric tests were applied. Effect sizes were reported in terms of partial Eta-Squared (${\eta }_{p}^{2}$), *r*, Cohen’s *d* [41], rank eta squared (η^2^(H)) and rank epsilon squared (ε^2^(R)). To account for multiple comparisons, e.g. in post-hoc testing, the Bonferroni correction was applied. Discriminative ability of both DDK tasks (i.e., AMR and SMR) and their combination to distinguish between HD and HC were examined by performing a logistic regression with leave-one-out cross-validation and calculating the receiver operating characteristic (ROC) curve. ROC curves for AMR, SMR and their combination were obtained by combined DDK rate and DDK irregularity measures. The area under the curve (AUC) was reported as an indicator of diagnostic accuracy. Further key parameters of sensitivity, specificity and accuracy were calculated for each ROC curve at the optimal cut-off point according to the Youden’s Index [42]. For HD patients, the relationship between oDDK parameters and clinical scores was examined using partial correlations (controlled for age and disease duration) with non-parametric spearman coefficient (due to violated normality assumption). The TMS score was further divided into sub-scores representing relevant motor domains including chorea, bradykinesia/rigidity, and dysarthria based on an exploratory factor analysis and clinical face validity. To explore the effect of ADMs on speech impairment, we applied explorative subgroup analysis using independent samples t-test or Mann-Whitney-U-test to compare oDDK rate and regularity in patients on and without ADMs. To examine dose-dependency non-parametric Kruskal–Wallis tests with patient group (HD-nADM vs. HD-ADM_low_ vs. HD-ADM_high_) as between-group factor followed by Bonferroni-corrected pairwise Mann–Whitney U post-hoc tests were conducted due to violation of normality assumption and/or variance inhomogeneity. In case of missing data, pairwise deletion (available-case analysis) was applied. The significance level was set to α = 0.05 for all analyses. R statistical programming language [43] and JASP (Version 0.16.1, [44]) were used for all statistical analyses.

## Results

### Clinical data

A total of 65 subjects participated in this study, 35 HD patients (18 men; age 22–76 years; *M* = 49.5 and *SD* = 12.1) and 30 healthy controls (HC; 14 men; age 27–78 years; *M* = 48.0 and *SD* = 13.9; Table 1).

**Table 1:** Descriptive sample characteristics of HD and HC

	HD (N = 35, 18 men)		HC (N = 30, 14 men)		p
	M (SD)	range (Median)	M (SD)	range (Median)	
Age (Y)	49.5 (12.1)	22–76 (52)	48.0 (13.9)	27–78 (53.5)	0.737^g^
Education (Y)	14.7 (3.57)	8–25 (14)	15.1 (4.29)	8–24 (15.5)	0.627^f^
Disease duration (Y)^a^	3.66 (2.34)	0.77–8.87 (3.41)	–	–	–
CAG repeats	44.8 (4.43)	39–58 (43)	–	–	–
CAP100^b^	106 (11.3)	77.7–129 (105)	–	–	–
Caudate Volume (mL)^c^	2.43 (0.52)	1.5–3.6 (2.4)	–	–	–
Putamen Volume (mL)^c^	4.17 (0.55)	2.9–5.1 (4.2)	–	–	–
Striatal Volume (mL)^c^	6.59 (0.84)	4.8–7.8 (6.5)	–	–	–
cUHDRS^d^	10.5 (3.01)	3.93–17.2 (10.4)	17.3 (1.56)	13.6–21.1 (17.3)	< 0.001***^f^
UHDRS-TMS	25.9 (15.3)	8–77 (25)	0.17 (0.91)	0–5 (0)	< 0.001***^g^
Dysarthria score	0.66 (0.54)	0–2 (1)	0.0 (0.0)	0–0 (0)	< 0.001***^g^
Bradykinesia/Rigidity	8.37 (5.63)	1–25 (8)	0.07 (0.37)	0–2 (0)	< 0.001***^g^
Chorea	7.14 (4.12)	1–16 (6)	0.0 (0.0)	0–0 (0)	< 0.001***^g^
UHDRS-TFC	10.7 (2.21)	3–13 (11)	13.0 (0.0)	13–13 (13)	< 0.001***^g^
UHDRS-IS	86.4 (11.9)	50–100 (90)	100 (0.0)	100–100 (100)	< 0.001***^g^
SDMT^e^	27.6 (10.6)	11–57 (27.5)	52.1 (11.1)	32–76 (53)	< 0.001***^f^
SDMT z-Score^e^	− 2.16 (1.01)	− 3.66–0.22 (− 2.3)	0.32 (0.79)	− 0.88–1.93 (0.21)	< 0.001***^f^

Abbreviations: *CAG* Cytosine-Adenine-Guanine; *cUHDRS* Composite UHDRS Score; *HC* Healthy controls; *HD* Huntington disease; *SDMT* Symbol Digit Modalities Test; *SDMT z-Score* z-standardized performance on SDMT [45]; *UHDRS* Unified Huntington's Disease Rating Scale; *UHDRS-IS* Independence Scale (ranging from 0-100%); *UHDRS-TFC* Total Functional Capacity (range 1–13, based on five questions concerning occupation, finances, domestic chores, activities of daily living, and care level); *UHDRS-TMS* Total Motor Score (range 0–124, based on ratings of 15 standardized examinations, e.g., oculomotor, dysarthria, chorea, dystonia, gait, and posture, evaluating the presence of HD motor features in a given subject); *Y* in years

^a^ analysis based on N = 33

^b ^CAP100 (CAG-Age-Product) = age x ([CAG – 30]/6.49) [32]

^c ^MRI volumetry analysis based on a subgroup of *N* = 19 HD patients in total; volumetric measures (caudate, putamen and striatal volume) from MR images were obtained using atlas-based volumetry (ABV)

^d^ Composite UHDRS Score was calculated for those patients in stage 1 and 2 (N = 33) in accordance with [46]; analysis based on N = 63 in total

^e^ analysis based on N = 64

^f^ independent samples t-Test (Students t-test or Welch Test depending on variance homogeneity tested using Levene Test) was used to test for significance of group differences

^g ^non-parametric Mann–Whitney-U-Test was used to test for significance of group differences due to violation of normality assumption tested using Shapiro-Wilks Test

* p < 0.05, ** p < 0.01, *** p < 0.001

All HD participants exhibited motor-signs but not all were functionally impaired at the time of study: 7 were in HD-ISS [30] stage 2, 28 in HD-ISS stage 3. Based on TFC scores [31], 22 were in TFC stage I, 11 in stage II and only 2 in stage III. A perceptual evaluation of dysarthria by raters well versed in assessing HD classified 12 patients as having normal speech (score = 0), 22 patients as displaying somewhat reduced intelligibility (score = 1, i.e., no need to repeat in order to be understood), and only 1 patient as exhibiting impaired speech (score = 2, i.e., must repeat to be understood). At the time of speech assessment, 16 patients (8 men; age 22–76 years; *M* = 51.0 and *SD* = 14.6) were on ADMs (HD-ADM); 19 HD participants (10 men; age 28–61 years; *M* = 48.3 and *SD* = 9.9) did not receive ADMs (HD-nADM). Details on the pharmacotherapy are given in the Supplement. As expected, patients on ADMs – contrasted with patients not receiving ADMs — displayed a somewhat more severe clinical phenotype, tended to have longer CAG repeat expansions and higher CAP100 scores [32] (Table 2). Also, in line with our expectations, participants with high doses of ADMs (HD-ADM_high_) had a shorter disease duration, tended to have longer CAG repeat expansions and tended to display more marked clinical signs, including more severe cognitive deficits, higher TMS scores and more pronounced striatal atrophy than patients with low doses of ADMs (HD-ADM_low_). Further details can be found in Supplementary Table 2 in the Supplement.

**Table 2:** Descriptive sample characteristics of HD patients with (HD-ADM) and without antidopaminergic medication (HD-nADM)

	HD-ADM (n = 16, 8 men)		HD-nADM (n = 19, 10 men)		p
	M (SD)	range (Median)	M (SD)	range (Median)	
Age (Y)	51.0 (14.6)	22–76 (52.5)	48.3 (9.86)	28–61 (51)	0.523^f^
Education (Y)	14.2 (4.02)	8–25 (13.5)	15.1 (3.21)	10–22 (14)	0.484^f^
Disease duration (Y)^a^	4.21 (2.72)	1.23–8.87 (3.64)	3.26 (2.01)	0.77–8.71 (3.41)	0.321^g^
CAG repeats	45.3 (5.67)	39–58 (43)	44.3 (3.15)	41–53 (43)	1.00^g^
CAP100^b^	109 (12.5)	86.9–129 (112)	102 (9.49)	77.7–121 (102)	0.069^f^
Caudate Volume (mL)^c^	2.36 (0.58)	1.5–3.6 (2.36)	2.49 (0.48)	1.9–3.17 (2.49)	0.584^f^
Putamen Volume (mL)^c^	4.34 (0.47)	3.7–5.1 (4.2)	4.02 (0.60)	2.9–4.65 (4.23)	0.208^f^
Striatal Volume (mL)^c^	6.69 (0.69)	5.9–7.8 (6.6)	6.50 (0.99)	4.8–7.8 (6.5)	0.639^f^
cUHDRS^d^	9.19 (2.46)	3.93–13.8 (9.05)	11.6 (3.05)	4.92–17.2 (12.0)	0.020*^f^
UHDRS-TMS	30.8 (17.9)	9–77 (30.5)	21.8 (11.7)	8–46 (21)	0.076^g^
Dysarthria score	0.69 (0.60)	0–2 (1)	0.63 (0.50)	0–1 (1)	0.862^g^
Bradykinesia/Rigidity	10.1 (5.71)	3–25 (9)	6.95 (5.30)	1–23 (5)	0.025*^g^
Chorea	7.88 (3.90)	2–14 (7.5)	6.53 (4.30)	1–16 (6)	0.325^g^
UHDRS-TFC	10.2 (2.35)	3–13 (11)	11.2 (2.06)	6–13 (12)	0.129^g^
UHDRS-IS	82.5 (12.4)	50–100 (85)	89.7 (10.6)	65–100 (90)	0.044*^g^
SDMT^e^	23.3 (8.21)	11–39 (26)	31.1 (11.3)	11–57 (32)	0.033*^f^
SDMT z-score^e^	− 2.44 (0.92)	− 3.66 – −0.4 (− 2.83)	− 1.94 (1.05)	− 3.55 – 0.22 (− 2.22)	0.160^f^
CPZ equivalent (mg/day)	210 (147)	75–600 (169)	0.0 (0.0)	0–0 (0.0)	–

Abbreviations: *CAG* Cytosine-Adenine-Guanine; *CPZ* chlorpromazine equivalents; *cUHDRS* Composite UHDRS Score; *HC* Healthy Controls; *HD* Huntington disease; *SDMT* Symbol Digit Modalities Test; *SDMT z-Score* z-standardized performance on SDMT [45]; *UHDRS* Unified Huntington Disease Rating Scale; *UHDRS-IS* Independence Score (ranging from 0 to 100%); *UHDRS-TFC* Total Functional Capacity (range 1–13, based on five questions concerning occupation, finances, domestic chores, activities of daily living, and care level); *UHDRS-TMS* Total Motor Score (range 0–124, based on ratings of 15 standardized examinations, e.g., oculomotor, dysarthria, chorea, dystonia, gait and posture, evaluating the presence of HD motor features in a given subject); *Y* in years

^a^ analysis based on N = 33

^b^ CAP100 (CAG-Age-Product) = age x ([CAG – 30]/6.49) [32]

^c ^MRI volumetry analysis based on a subgroup of N = 19 HD patients in total, i.e., n = 10 in HD-nADM and n = 9 in HD-ADM, respectively; volumetric measures (caudate, putamen and striatal volume) from MR images were obtained using atlas-based volumetry (ABV)

^d ^Composite UHDRS Score was calculated for those patients in stage 1 and 2 (N = 33) in accordance with [46]

^e^ analysis based on N = 34;

^f ^ independent samples t-Test (Students t-test or Welch Test depending on variance homogeneity tested using Levene Test) was used to test for significance of group differences

^g ^ non-parametric Mann–Whitney-U-Test was used to test for significance of group differences due to violation of normality assumption tested using Shapiro-Wilks Test

* p < 0.05, ** p < 0.01, *** p < 0.001.

**Fig. 1:**
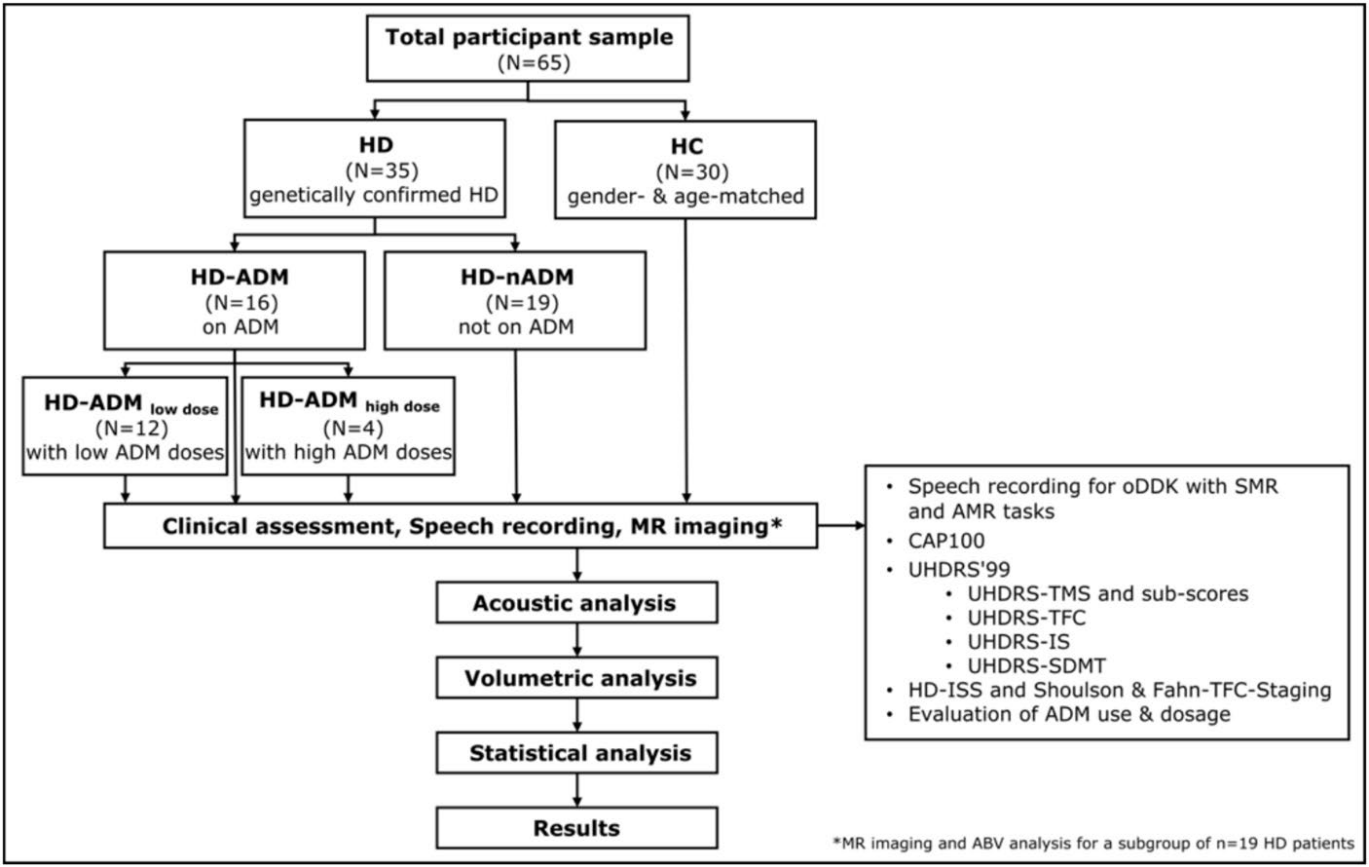
Study flowchart. Abbreviations: *ABV* atlas-based volumetry; *ADM * antidopaminergic medication; *AMR* alternating motion rate; *CAP* CAG-Age-product; o*DDK* oral diadochokinesis; *HD* Huntington disease; *HC* healthy controls; *HD-ADM* HD subjects with antidopaminergic medication; *HD-nADM* HD subjects without antidopaminergic medication; *HD-ISS* Huntington disease international staging system; *MR imaging* magnetic resonance imaging; *N* number of participants; *SMR* sequential motion rate; *SDMT* Symbol Digit Modalities Test; *UHDRS* Unified Huntington's Disease Rating Scale; *UHDRS-IS* Independence Scale; *UHDRS-TFC* Total Functional Capacity; *UHDRS-TMS* Total motor score

For the HC group, the standard perceptual examination of dysarthria showed speech within normal limits for all controls (score = 0). There were no relevant differences between HD and HC regarding their demographic characteristics (Table 1). Figure 1 provides an overview of the study design.

### Comparison of oDDK parameters in HD and HC

**Fig. 2:**
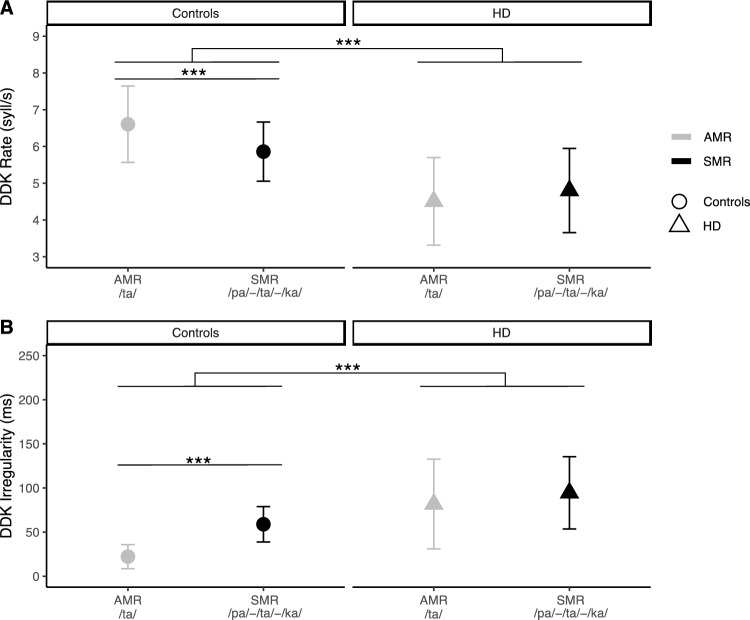
Analysis for group differences between HD patients (HD) and controls (HC) showed that **A** HD patients were slower in repeating /ta/ (AMR) and /pa/-/ta/-/ka/ (SMR; i.e., DDK rate) than HC. While HC were slower in SMR than in AMR, HD patients showed no difference in the repetition rate for AMR and SMR. **B** HD patients were more irregular (i.e., DDK irregularity) than HC in repeating /ta/ (AMR) and /pa/-/ta/-/ka/ (SMR). While the performance of HD patients was similar in both tasks, AMR and SMR, HC showed more irregularities in repeating /pa/-/ta/-/ka/ (SMR) than /ta/ (AMR). Abbreviations: *AMR* alternating motion rate, *oDDK* oral diadochokinesis, *HC* healthy controls, *HD* Huntington disease, *SMR* sequential motion rate, *syll/s* number of syllable vocalizations per second. Analysis using repeated-measures ANOVA with between-group factor (HD vs. HC) and within-group factor (AMR vs. SMR); Post-hoc analysis using t-tests with Bonferroni-correction for multiple comparisons; * p < 0.05, ** p < 0.01, *** p < 0.001. Symbols represent mean values and error bars represent standard deviation

The acoustic analysis results for oDDK rate and regularity in HD and HC for AMR (/ta/) and SMR (/pa/-/ta/-/ka/) are shown in Fig. 2. Overall, HD scored worse in all oDDK parameters compared to HC.

More specifically, the repeated-measures ANOVA for oDDK rate showed a significant main effect for GROUP, *F*(1,63) = 44.2, p < 0.001, ${\eta }_{p}^{2}$ = 0.412, indicating that HD patients were significantly slower in repeating /ta/ and /pa/-/ta/-/ka/ than HC. There was no significant main effect for TASK, *F*(1,63) = 3.66, p = 0.060, ${\eta }_{p}^{2}$ = 0.055, indicating that oDDK rate did not differ between AMR and SMR tasks. There was, however, an interaction effect for GROUP × TASK, *F*(1,63) = 19.3, p < 0.001, ${\eta }_{p}^{2}$ = 0.234. Post-hoc analyses suggest that the interaction between GROUP and TASK was significant, because HC were significantly slower in repeating /pa/-/ta/-/ka/ compared to /ta/ (p < 0.001), whereas HD patients were equally slow in both tasks (p = 0.147).

Regarding oDDK regularity, we detected a significant main effect for GROUP, *F*(1,63) = 37.4, p < 0.001, ${\eta }_{p}^{2}$ = 0.373, indicating that HD patients were more irregular in repeating /ta/ and /pa/-/ta/-/ka/ than HC. There was also a significant main effect for TASK, *F*(1,63) = 32.0, p < 0.001, ${\eta }_{p}^{2}$ = 0.337, indicating that oDDK irregularity was more marked for SMR compared to AMR. We further detected a significant interaction effect for GROUP × TASK, *F*(1,63) = 7.5, p = 0.008, ${\eta }_{p}^{2}$ = 0.107. Post-hoc analyses showed that the interaction between GROUP and TASK was significant, because HC were significantly more irregular in repeating /pa/-/ta/-/ka/ (SMR) compared to /ta / (AMR; p < 0.001), whereas task performance was similar in HD (p = 0.072).

### Accuracy of oDDK tasks in differentiating HD patients and HC

ROC curves showed for both oDDK tasks (individually and combined) a high potential to discriminate between HD and HC (AUC > 80.0%; see Supplementary Material Fig. 1 and Supplementary Material Table 3). AMR discriminated best between HD and HC in terms of AUC = 95.0% (95%-CI [90.1; 99.8%]). Combining AMR with SMR did not increase diagnostic accuracy (AUC = 95.0%, 95%-CI [90.1; 99.8%]). The lowest discriminative accuracy was detected for SMR alone (AUC = 84.3%, 95%-CI [74.1; 94.5%]). Leave-one-out cross-validation showed a good predictive performance for all logistic regression models and was best for AMR alone (Accuracy: 86.2%), second best for combined AMR and SMR (Accuracy: 84.6%) and worst for SMR alone (Accuracy: 78.5%).

**Table 3:** Partial correlations between parameters of oDDK and clinical scales (controlling for the influence of age and disease duration) for the HD group

	CAP100^a^	Caudate Volume^b^	Putamen Volume^b^	Striatal Volume^b^	cUHDRS^c^	UHDRS-TMS^a^	UHDRS-Dysarthria^a^	UHDRS-Bradykinesia/ Rigidity^a^	UHDRS-Chorea^a^	UHDRS-TFC^a^	SDMT^d^	SDMT z-Score^d^
oDDK rate												
AMR (/ta/)	– 0.150 (p = 0.420)	0.604* (p = 0.017)	0.126 (p = 0.655)	**0.512** (p = 0.051)	0.508** (p = 0.005)	– 0.433* (p = 0.015)	− 0.418* (p = 0.019)	– 0.588*** (p < 0.001)	0.051 (p = 0.785)	0.488** (p = 0.005)	0.646*** (p < 0.001)	0.509** (p = 0.004)
SMR (/pa/-/ta/-/ka/)	– 0.091 (p = 0.626)	0.455 (p = 0.088)	0.237 (p = 0.396)	**0.514** (p = 0.050)	0.497** (p = 0.006)	– 0.428* (p = 0.016)	– 0.343 (p = 0.059)	− 0.451* (p = 0.011)	0.00 (p = 0.997)	0.402* (p = 0.025)	0.400* (p = 0.029)	0.419* (p = 0.021)
oDDK regularity												
AMR (/ta/)	0.131 (p = 0.481)	– 0.714** (p = 0.003)	– 0.377 (p = 0.165)	– 0.734** (p = 0.002)	– 0.478** (p = 0.009)	0.519** (p = 0.003)	0.466** (p = 0.008)	0.433* (p = 0.015)	0.371* (p = 0.040)	– 0.419* (p = 0.019)	– 0.466** (p = 0.009)	– 0.463** (p = 0.010)
SMR (/pa/-/ta/-/ka/)	0.052 (p = 0.780)	– 0.560* (p = 0.030)	– 0.354 (p = 0.195)	– 0.605* (p = 0.017)	– 0.227 (p = 0.235)	0.31 (p = 0.090)	0.322 (p = 0.077)	0.36* (p = 0.047)	0.007 (p = 0.971)	– 0.313 (p = 0.087)	– 0.248 (p = 0.187)	– 0.273 (p = 0.144)

Abbreviations: *AMR* alternating motion rate;* CAP* CAG-Age-Product; *cUHDRS* Composite UHDRS*; oDDK* oral diadochokinesis; *SDMT* Symbol Digit Modalities Test; *SDMT z-score* z-standardized performance on SDMT; *SMR* sequential motion rate; *UHDRS* Unified Huntington’s Disease rating scale; *UHDRS-TMS* Total Motor Score (range 0–124, based on ratings of 15 standardized examinations, e.g., oculomotor, dysarthria, chorea, dystonia, gait and posture, evaluating the presence of HD motor features in a given subject); UHDRS-Bradykinesia/Rigidity subscale includes the following items: Pro-/Supination L/R, Finger Taps L/R, Bradykinesia-Body, Rigidity L/R, Gait, Tandem Walking (possible range: 0 to 36); UHDRS-Chorea includes the following items: Chorea Trunk, BOL, Face, Upper and Lower Extremities L/R, Tongue Protrusion (possible range: 0 to 32); UHDRS-Dysarthria includes the dysarthria item (possible range: 0 to 4); *UHDRS-TFC* Total Functional Capacity (possible range: 1–13); interpretation according to Cohen (1988, pp. 79–80) [41]: r < 0.1 may be interpreted as no effect; 0.1 ≤ r < 0.3 indicates a small effect size; 0.3 ≤ r < 0.5 refers to a moderate effect size; r ≥ 0.5 is equivalent to a strong effect size (written in bold);

* p < 0.05, ** p < 0.01, *** p < 0.001

^a^ analysis based on N = 33 observations of HD patient group

^b^ analysis for Caudate Volume, Putamen Volume and Striatal Volume (MRI volumetry data) based on N = 17 observations of HD patient group

^c^ analysis based on N = 31 observations of HD patient group

^d^ analysis based on N = 32 observations of HD patient group

### Correlation of speech parameters to clinical scores and extent of striatal atrophy

Pairwise partial correlations between oDDK parameters, clinical scores and biological indicators of disease severity were conducted for manifest patients (HD group) to investigate the relation between oDDK and disease severity while controlling for disease duration and age (see Table 3).

For oDDK rate, we detected multiple significant partial correlations with clinical scores and striatal atrophy for both AMR and SMR, with higher coefficients for AMR compared to SMR. The strongest correlations were detected for SDMT, caudate volume, bradykinesia/rigidity and the composite UHDRS.

For oDDK irregularity, we again found multiple significant partial correlations with clinical scores and with striatal atrophy for both AMR and SMR, with higher coefficients for AMR. The strongest correlations were observed with striatal atrophy and the severity of the motor phenotype (UHDRS-TMS).

### Explorative subgroup analysis on the impact of antidopaminergic medication (ADM)

**Fig. 3:**
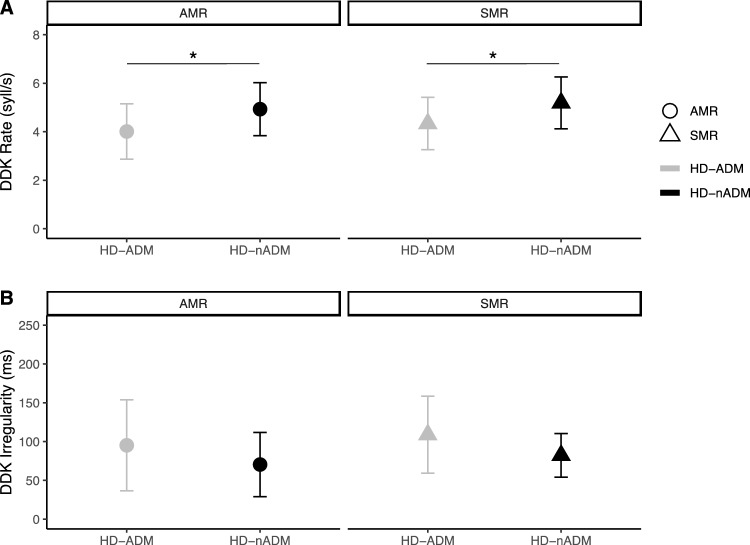
Exploratory subgroup analysis for group differences between HD patients with (HD-ADM) and without antidopaminergic medication (HD-nADM) showed that **A** HD patients on antidopaminergic medication (HD-ADM) were slower in repeating /ta/ (AMR) and /pa/-/ta/-/ka/ (SMR; i.e., DDK rate) than patients without antidopaminergic medication (HD-nADM) but **B** they were not significantly more irregular in their ability to repeat /ta/ (AMR) and /pa/-/ta/-/ka/ (SMR; i.e., DDK irregularity). Abbreviations: *AMR* alternating motion rate, *oDDK* oral diadochokinesis, *HD* Huntington disease, *SMR* sequential motion rate, *syll/s* number of syllable vocalizations per second. Analysis using t-tests or Mann-Whitney-U-test with Bonferroni-correction for multiple comparisons; * p < 0.05. Symbols represent mean values and error bars represent SD of the mean

In a subgroup analysis, we compared 16 patients on ADMs (HD-ADM) with 19 patients not receiving ADMs (HD-nADM) with respect to oDDK performance. Patients on ADMs displayed slower oDDK rates than patients not receiving ADMs (see Fig. 3) in both AMR (p = 0.021, *|d|* = 0.83) and SMR tasks (p = 0.026, |*d|* = 0.79). Patients on ADMs tended to be more irregular than those not receiving ADMs in both tasks; however, there were no statistically significant differences in oDDK irregularity, neither for AMR (p = 0.154) nor for SMR (p = 0.152). Compared to HC, both subgroups showed slower oDDK rates and higher irregularities in both tasks with patients on ADMs showing more pronounced differences to HC than patients without ADMs.

**Fig. 4:**
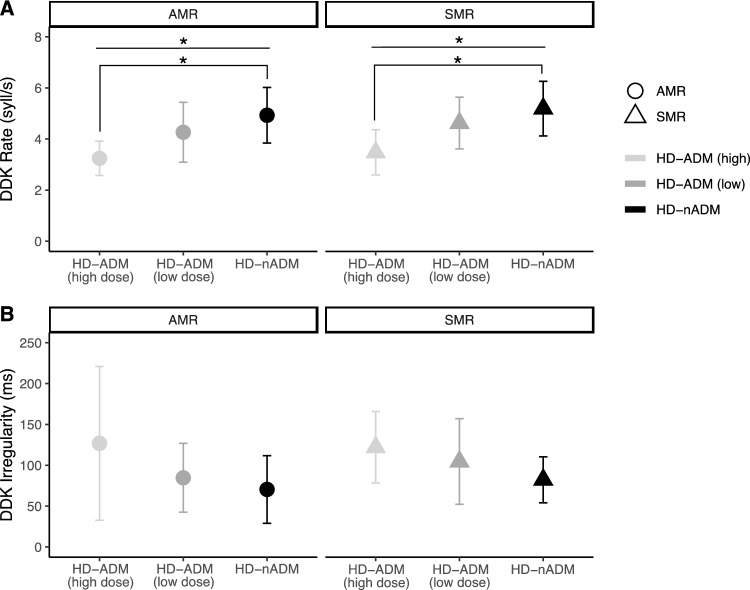
Exploratory subgroup analysis for group differences between HD patients with high doses (HD-ADM_high_), low doses (HD-ADM_low_) and without antidopaminergic medication (HD-nADM) showed that **A** patients with high doses of antidopaminergic medication (HD-ADM_high_) were slower in repeating /ta/ (AMR) and /pa/-/ta/-/ka/ (SMR; i.e., DDK rate) than patients without antidopaminergic medication (HD-nADM) **B** but were not more irregular in repeating /ta/ (AMR) and /pa/-/ta/-/ka/ (SMR; i.e., DDK irregularity). Abbreviations: *AMR* alternating motion rate, *oDDK* oral diadochokinesis, *HD* Huntington disease, *SMR* sequential motion rate, *syll/s* number of syllable vocalizations per second. Analysis using Kruskal–Wallis tests with patient group (HD-nADM vs. HD-ADM_low_ vs. HD-ADM_high_) as between-group factor followed by pairwise Mann–Whitney-U-post-hoc tests with Bonferroni-correction for multiple comparisons; * p < 0.05. Symbols represent mean values and error bars represent SD of the mean

After stratification of the HD-ADM cohort into low (HD-ADM_low_) and high dosage groups (HD-ADM_high_), we performed Kruskal–Wallis tests to investigate ADM dosage effects on oDDK. HD-ADM_high_ patients tended to be slower than HD-ADM_low_ patients who in turn tended to be slower than HD-nADM patients, i.e. for oDDK rate, we found a significant main effect of GROUP for both tasks: AMR (χ^2^(2) = 7.73, p = 0.021, ε^2^(R) = 0.23, η^2^(H) = 0.25) and SMR (χ^2^(2) = 8.43, p = 0.015, ε^2^(R) = 0.25, η^2^(H) = 0.20). Post-hoc testing with Bonferroni-corrected pairwise Mann–Whitney-U-tests showed that only the difference between patients not receiving ADMs and patients with high ADM dosages was significant for both tasks (AMR: p = 0.012, large effect size *r* = 0.56; SMR: p = 0.018, large effect size *r* = 0.54). For oDDK irregularity, there was no significant main effect of GROUP for neither task (AMR: χ^2^(2) = 1.76, p = 0.414; SMR: χ^2^(2) = 3.27, p = 0.195; see Fig. 4).

## Discussion

This study investigated the question whether challenging or less challenging oral diadochokinetic (oDDK) tasks, SMR and AMR, respectively, are better suited to objectively detect speech alterations in manifest HD patients with little or moderate functional impairment, i.e., in early-to-moderate TFC-stages. Our study disclosed that the performance in the less challenging oDDK task (AMR) distinguishes particularly well between HD and HC (AUC = 95.0%), correlates well with clinical indicators of disease severity as well as with the extent of striatal atrophy as assessed by MR-imaging and ABV. In addition, HD patients on ADMs displayed slower oDDK rates but unchanged oDDK irregularities, suggesting that a quantitative analysis of oDDK is sensitive to pharmacological interventions. Given the correlation with indicators of disease severity, our data suggest that a quantitative assessment of oDDK has potential as progression biomarker and may assist in assessing the impact of pharmacological interventions in HD.

This study confirms prior observations that HD patients show slower oDDK rates [14, 20] and higher oDDK irregularities [14, 21] than HC, independent of the oDDK task employed (i.e., AMR and SMR, respectively).

HCs showed slower oDDK rates and more oDDK irregularities in the more challenging oDDK task, SMR, compared to their performance in the less demanding oDDK task, AMR. In contrast, in HD a different pattern was observed: patients showed decreased oDDK rates and more oDDK irregularities in both tasks. The physiological difference in performance between the less challenging task (AMR) and the more demanding task (SMR) observed in HC was no longer detectable in HD patients at this stage of the disease process, i.e., HD-ISS stages 2 and 3 [30]. To the best of our knowledge, there are no prior studies directly comparing AMR and SMR performance in HD. Our result is in line with findings suggesting that HD patients show more pronounced deficits in seemingly simple, repeated, overlearned, ‘automated’ tasks (i.e., reading words in the Stroop Word Reading Test) than in tasks with seemingly higher demands on executive functions (i.e., naming the colour of the ink in which a colour name is written instead of reading a name of a colour in the Stroop test interference condition) [47].

There were clear associations of oDDK performance with various indicators of disease severity (e.g., motor, cognitive and functional impairments) related to striatal dysfunction. The ability to keep a regular pace and rhythm in producing alternating and sequential syllables is thought to dependent on the functioning of subcortical networks and their overall integrity [19, 48, 49]. As the striatum is involved not only in motor control and execution [50], but also in the speech production process [6], including selection [51], sequencing, motor programming [52], initiation [6, 53] and execution [6], striatal dysfunction may directly cause disturbances in those aspects of speech production and result in abnormal speech features in HD. Our study showed moderate to strong correlations of oDDK measures to motor features, namely the UHDRS-TMS sub-score bradykinesia/rigidity. The bradykinesia/rigidity sub-score correlated to the altered oDDK parameters rate and regularity in both tasks, i.e., AMR and SMR. This is in line with previous findings where HD patients presenting with more severe bradykinesia and rigidity displayed slower and less steady syllable repetition [21]. Interestingly, for the sub-score chorea, only an AMR task performance (irregularity), but not the SMR task performance showed significant correlations to chorea sub-score. Similarly, strong correlations were found between oDDK rates and SDMT scores, with slower oDDK rates being associated with worse cognitive scores. This is in line with previous research showing correlations of different speech parameters (e.g., pause ratio, speech rate, and syllable repetition capacity) with cognitive scores [21]. Lastly, there was a trend towards a correlation between reduced oDDK speed and regularity and lower TFC scores, in line with a previous study [21]. Of note, oDDK performance in AMR tasks yielded a larger number of statistically significant correlations to clinical measures of HD severity than SMR tasks: e.g., for oDDK regularity, 8 out of 8 clinical parameters correlated significantly to AMR task performance compared with 1 out of 8 for SMR task performance (see Table 3).

Biological measures of HD severity, i.e., the extent of atrophy in caudate, putamen and striatum overall correlated well with oDDK measures, with caudate atrophy being more predictive than putaminal atrophy. Again, correlations with performance in the AMR task tended to be stronger than in the SMR task.

Regarding the impact of ADMs on speech, our study shows for the first time a significantly slower DDK rate for both AMR and SMR tasks in manifest HD patients on ADMs compared to HD patients not receiving ADMs. This is in line with previous studies detecting a certain slowing of articulation speed and timing across different speech parameters [29] which may reflect an overall slowing of voluntary movements in HD patients on ADMs as demonstrated by van Vugt et al. [26]. Interestingly, we did not find an increase of oDDK irregularity in HD patients on ADMs compared to those not receiving ADMs which may suggest that antidopaminergic medications do not lead to a reduced control over the rhythm and regularity of intentional (speech) movements, but primarily to an undesirable slowing of intentional (speech) movements. In addition, our results provide preliminary support for the hypothesis that this undesirable slowing of articulation speed by antidopaminergic pharmacotherapy may be dose dependent. We observed that patients with higher doses of ADMs (HD-ADM_high_) showed significantly slower DDK rates for both tasks compared to patients without ADMs (HD-nADM), while oDDK rates (again for both tasks) for patients on low dosages of ADMs (HD-ADM_low_) did not significantly differ from those of patients without ADMs (HD-nADM). We did not find a dose-dependent increase of oDDK irregularity in HD patients on ADMs further supporting the hypothesis that ADMs do not lead to a reduced control over the regularity of intentional (speech) movements. Due to the observational design of our study, however, it is not possible to establish the root cause of the worsening bradykinesia. The more pronounced bradykinesia may truly be side effects of ADMs or may reflect a worsening phenotype, i.e., be a part of the natural history of HD: patients on higher dosages of ADMs had more expanded CAG repeats, higher CAP100 values and more pronounced striatal atrophy.

Here we provide for the first time evidence that performance in the AMR task separate manifest HD in early clinical stages from HC particularly well. Combining AMR with SMR measures did not improve discrimination further. This finding is in line with similar observations in patients with spastic dysarthria [11]. We suggest, therefore, that in manifest HD patients in early/moderate clinical stages it is sufficient to assess oDDK simply employing the AMR task without compromising the ability to detect HD-related speech alterations.

## Limitations

The main limitations of the present study are the cross-sectional, observational study design, the relatively small sample size and the limited dosage range of ADMs investigated. In addition, a limitation of the study is that — due to pandemic restrictions at that time — speech recording and MRI could not always be performed at the same time, thus blurring the correlation between MRI features and the quantitative analysis of speech recordings. However, despite the temporal gaps, MRI allowed for proper HD-ISS staging. Considering our encouraging results, future work should explore quantitative acoustic analysis of speech as biomarker for disease progression in a longitudinal study design and to track oDDK over an extended disease course and to study the impact of ADM in a larger sample of HD patients (e.g., greater dosage range of ADMs) in a serial fashion (e.g., before and after starting ADMs and following adjustments of dosage) to establish possible dose–effect relationships.

## Conclusion

This study shows that a single, not challenging oDDK task, AMR, is sensitive and sufficient to capture changes in rate and regularity of oDDK in early-to-moderate manifest HD. This less challenging oDDK task seems to reflect the severity of the clinical phenotype and the degree of striatal atrophy and may be sensitive to the effects of ADMs. These findings further support the potential of quantitative acoustic speech analysis to serve as an objective biomarker for monitoring disease progression and may point to a role in assessing the impact of therapeutic interventions.

## Supplementary Information

Below is the link to the electronic supplementary material.

Supplementary file1 (DOCX 132 KB)

## Data Availability

The data that support the findings of this study are available from the corresponding author on reasonable request. The data are not publicly available due privacy or ethical restrictions.
