# Supplementary material for: Challenging or less challenging oral diadochokinetic tasks—what works best in Huntington disease? A cross-sectional study
Source: J Neurol. 2025 Oct 15;272(10):697. doi: 10.1007/s00415-025-13310-x (PMC12528184; doi:10.1007/s00415-025-13310-x)
Supplement: Supplementary file 1 — Supplementary file1 (DOCX 132 KB) [file 415_2025_13310_MOESM1_ESM.docx]

**Challenging or less challenging oral diadochokinetic tasks - what works best in Huntington disease? A cross-sectional study**

Wiebke Hannemann^1^, Lukas Stahuber^1^, Tomas Kouba^2^, Katrin S. Lindenberg^1^, Daniel Rapp^1^, Jan Lewerenz^1^, Hans-Jürgen Huppertz^3^, Tereza Tykalova^2^, Jan Rusz^2^, G. Bernhard Landwehrmeyer^1^*, Alzbeta Mühlbäck^1,4,5^

^1^ Huntington Center Ulm (HCU), Department of Neurology, Ulm University, Ulm, Germany

^2^ Department of Circuit Theory, Faculty of Electrical Engineering, Czech Technical University in Prague, Prague, Czech Republic

^3^ Swiss Epilepsy Clinic, Klinik Lengg, Zürich, Switzerland

^4^ Huntington Center South, kbo-Isar-Amper-Klinikum, Taufkirchen, Germany

^5^ Department of Neurology and Centre of Clinical Neuroscience, First Faculty of Medicine, Charles University

and General University Hospital, Prague, Czech Republic

***Corresponding author address:**


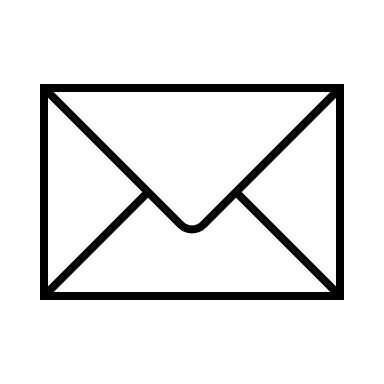
Prof. G. Bernhard Landwehrmeyer, MD, FRCP

Department of Neurology

Oberer Eselsberg 45, 89081 Ulm

Ulm University, Germany

bernhard.landwehrmeyer@uni-ulm.de

Supplementary Material

## Clinical data regarding of antidopaminergic medication (ADM)

To explore the impact of antidopaminergic medication (ADM) on oDDK parameters, the HD patient group was divided into patients on ADMs (HD-ADM) and patients not receiving ADMs (HD-nADMs). Patients with ADMs were either treated with monotherapy or with a combination of drugs. The HD-ADM group was further stratified into a low (HD-ADM_low_) and high dosage group (HD-ADM_high_) by an experienced neurologist to allow the investigation of a potential dose-dependency effect. Please see Supplementary Table 1 below for an overview of the medication included and of the classification into low and high doses.

**Supplementary Table 1.** List of included antidopaminergic medication (ADM) and of classification into low and high doses.

| **Drug** | ***N*** | ***low doses***  ***(HD-ADM_low_)*** | ***high doses***  ***(HD-ADM_high_)*** |
| --- | --- | --- | --- |
| Olanzapine | 7 | ≤10 mg | ≥ 15 mg |
| Tiapride | 6 | ≤ 400 mg | ≥ 400 mg |
| Aripiprazole | 1 | 5 mg | - |
| Tiapride + Tetrabenazine | 1 | - | 300 mg / 37,5 mg |
| Olanzapine + Quetiapine | 1 | 2,5 mg / 37,5 mg | - |

## Baseline demographic results for the HD-ADM_high_ and HD-ADM_low_ subgroup

**Supplementary Table 2.** Descriptive sample characteristics of subgroup of HD patients with low doses (HD-ADM_low_) and high doses of antidopaminergic medication (HD-ADM_high_)

|  | HD-ADM_low_ (*n*=12, 6 men) | | HD-ADM_high_ (*n*=4, 2 men) | | *p* |
| --- | --- | --- | --- | --- | --- |
|  | *M* (SD) | range (Median) | *M* (SD) | range (Median) |  |
| Age (Y) | 53.9 (14.6) | 22–76 (56.5) | 42.2 (12.3) | 31–58 (40) | .127^f^ |
| Education (Y) | 14.7 (4.52) | 8–25 (14) | 12.8 (1.50) | 11–14 (13) | .381^f^ |
| Disease duration (Y)^a^ | 5.25 (2.53) | 1.97–8.87 (4.96) | 1.59 (0.32) | 1.23–1.93 (1.61) | .002^f^ |
| CAG repeats | 44.2 (5.10) | 39–58 (42.5) | 48.8 (6.65) | 43–55 (48.5) | .201^f^ |
| CAP100^b^ | 108 (12.1) | 86.9–129 (109) | 113 (14.7) | 92.1–126 (118) | .521^f^ |
| Caudate Volume (mL)^c^ | 2.50 (0.55) | 1.8–3.6 (2.40) | 1.85 (0.49) | 1.5–2.2 (1.85) | .111^f^ |
| Putamen Volume (mL)^c^ | 4.41 (0.47) | 3.84–5.1 (4.2) | 4.10 (0.57) | 3.7–4.5 (4.1) | .639^f^ |
| Striatal Volume (mL)^c^ | 6.90 (0.63) | 6.1–7.8 (6.7) | 5.95 (0.07) | 5.9–6.0 (5.95) | .056^f^ |
| cUHDRS^d^ | 9.65 (2.23) | 6.63–13.8 (9.57) | 7.36 (2.97) | 3.93–9.27 (8.87) | .365^f^ |
| UHDRS-TMS | 24.8 (10.2) | 9.0–38.0 (27.5) | 48.5 (25.8) | 22.0–77.0 (47.5) | .110^f^ |
| Dysarthria score | 0.58 (0.51) | 0–1 (1) | 1.00 (0.82) | 0–2 (1) | .352^f^ |
| Bradykinesia/Rigidity | 8.0 (2.49) | 3–11 (9) | 16.2 (8.50) | 8–25 (16) | .093^f^ |
| Chorea | 7.50 (4.27) | 2–14 (6.5) | 9.00 (2.58) | 6–12 (9) | .534^f^ |
| UHDRS-TFC | 10.8 (1.27) | 9–13 (11) | 8.50 (4.04) | 3–12 (9.5) | .367^f^ |
| UHDRS-IS | 86.7 (6.51) | 80–100 (85) | 70.0 (18.3) | 50–90 (70) | .102^f^ |
| SDMT^e^ | 25.1 (8.06) | 14–39 (26) | 16.3 (4.73) | 11–20 (18) | .128^f^ |
| SDMT z-score^e^ | -2.22 (0.90) | -3.45– -0.4 (-2.28) | -3.30 (0.31) | -3.66– -3.1 (-3.14) | .031^f^ |
| CPZ equivalent (mg/day) | 148 (72.7) | 75–300 (131) | 394 (166) | 225–600 (375) | .005^f^ |
| *Abbreviations:* *CAG* Cytosine-Adenine-Guanine; *CAP* CAG-Age-Product; *CPZ* chlorpromazine equivalents; *cUHDRS* Composite UHDRS Score; *HC* Healthy Controls; *HD* Huntington disease; *SDMT* Symbol Digit Modalities Test; *SDMT z-Score* z-standardized performance on SDMT [44]; *UHDRS* Unified Huntington Disease Rating Scale; *UHDRS-IS* Independence Score (ranging from 0 to 100 %); *UHDRS-TFC* Total Functional Capacity (range 1–13, based on five questions concerning occupation, finances, domestic chores, activities of daily living, and care level); *UHDRS-TMS* Total Motor Score (range 0–124, based on ratings of 15 standardized examinations, e.g., oculomotor, dysarthria, chorea, dystonia, gait and posture, evaluating the presence of HD motor features in a given subject); *Y* in years  *Notes:*  ^a^ Analysis based on N=14  ^b^ CAP100 = age x ([CAG – 30] / 6.49) [31]  ^c^ volumetric measures (caudate, putamen and striatal volume) from MR images were obtained using atlas-based volumetry (ABV) with MRI volumetry analysis based on a subgroup of N=9 HD patients (in the HD-ADM subgroup with N=2 in the HD-ADM_high_ subgroup and N=7 in the HD-ADM_low_ subgroup);  ^d^ cUHDRS was calculated for those patients in stage 1 and 2 (for N=15 in this subgroup) in accordance with [43]  ^e^ analysis based on N=15  ^f^ non-parametric Mann-Whitney U-Test was used to test for significance of group differences due to violation of normality assumption tested using Shapiro-Wilks Test  * *p* < 0.05, ** *p* < 0.01, *** *p* < 0.001. | | | | | |

Supplementary information for the accuracy of oDDK tasks in differentiating HD patients and HC

**Supplementary Material Table 3** Key parameters calculated from ROC analysis for the optimal cut-off according to Youden’s

|  | **AUC** | **95%-CI** | **Sensitivity** | **Specificity** | **Accuracy** | **Youden’s Index** | **Threshold** |
| --- | --- | --- | --- | --- | --- | --- | --- |
| AMR | **95.0%*** | 90.1-99.8% | 85.7% | 96.7% | 90.7% | 1.82 | 0.38 |
| SMR | **84.3%** | 74.1-94.5% | 80.0% | 83.3% | 81.5% | 1.63 | 0.49 |
| Combined | **95.0%*** | 90.1-99.8% | 82.9% | 96.7% | 89.2% | 1.80 | 0.35 |
| *Abbreviations:* *AMR* alternating motion rate; *AUC* area under the curve in % (*test with the highest AUC); *SMR* sequential motion rates; *ROC* receiver operating characteristic; *95%-CI* 95% confidence interval (in %);  *Notes:* Youden’s Index = operationalizes the optimal cut-off as the threshold that maximizes the distance to the diagonal line (Youden’s = max(Sensitivities + Specificities)); Threshold = optimal cut-off according to Youden’s; Accuracy = (True Positive Count + True Negative Count) / N at the optimal cut-off according to Youden’s; Sensitivity = True positive count / (True Positive Count + False Negative Count) at the optimal cut-off according to Youden’s; Specificity = True Negative Count / (True Negative Count + False Positive Count) at the optimal cut-off according to Youden’s. | | | | | | | |

Supplementary Figures

**Supplementary Material Fig. 1** ROC curves obtained between HD and HC groups using a combination of oDDK rate and oDDK irregularity across both speaking tasks. *Abbreviations:* *AMR* alternating motion rate; *AUC* area under the curve, *oDDK* oral diadochokinesis, *HC* Healthy controls, *HD* Huntington disease, *ROC* receiver operating characteristic, *SMR* sequential motion rate

**
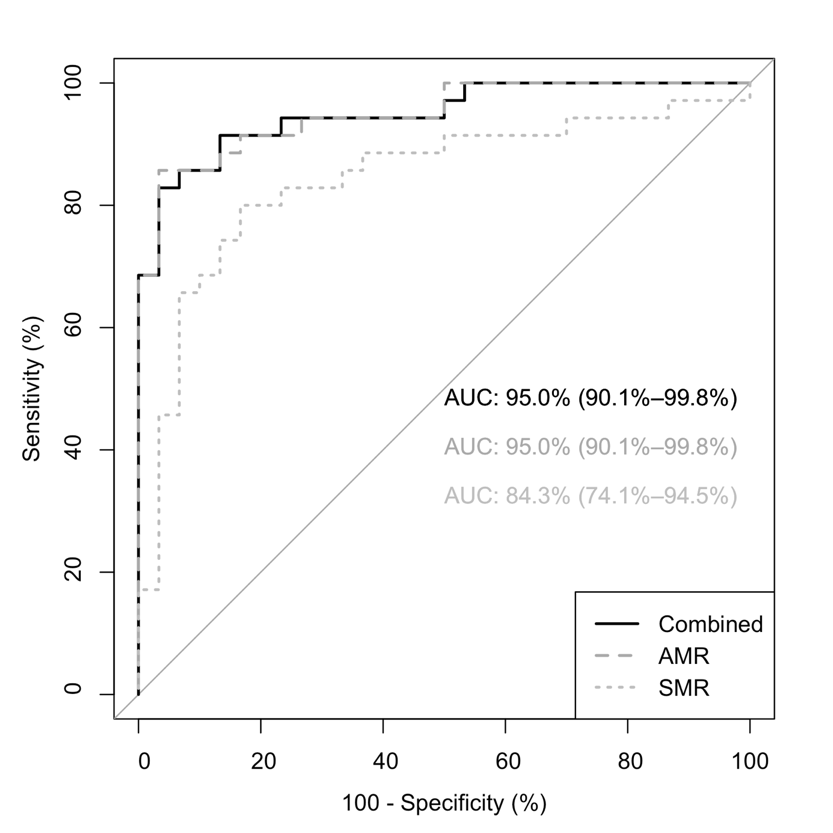
**
